# Deep learning CT reconstruction improves liver metastases detection

**DOI:** 10.1186/s13244-024-01753-1

**Published:** 2024-07-06

**Authors:** Achraf Kanan, Bruno Pereira, Constance Hordonneau, Lucie Cassagnes, Eléonore Pouget, Léon Appolinaire Tianhoun, Benoît Chauveau, Benoît Magnin

**Affiliations:** 1 Department of Radiology, Estaing Hospital, Clermont University Hospital, Clermont-Ferrand, France; 2 Department of Biostatistics, DRCI, Clermont University Hospital, Clermont-Ferrand, France; 3 Institut Pascal, UMR 6602 CNRS, Université Clermont Auvergne, Clermont-Ferrand, France; 4 Department of Radiology, Gabriel Montpied Hospital, Clermont University Hospital, Clermont-Ferrand, France; 5 Department of Radiology, Tengandogo’ Ouagadougou University Hospital Center, Ouagadougou, Burkina Faso; 6 DI2AM, DRCI, Clermont University Hospital, Clermont-Ferrand, France

**Keywords:** Liver neoplasm, Image reconstruction, Artificial intelligence, Deep learning, Computed tomography

## Abstract

**Objectives:**

Detection of liver metastases is crucial for guiding oncological management. Computed tomography through iterative reconstructions is widely used in this indication but has certain limitations. Deep learning image reconstructions (DLIR) use deep neural networks to achieve a significant noise reduction compared to iterative reconstructions. While reports have demonstrated improvements in image quality, their impact on liver metastases detection remains unclear. Our main objective was to determine whether DLIR affects the number of detected liver metastasis. Our secondary objective was to compare metastases conspicuity between the two reconstruction methods.

**Methods:**

CT images of 121 patients with liver metastases were reconstructed using a 50% adaptive statistical iterative reconstruction (50%-ASiR-V), and three levels of DLIR (DLIR-low, DLIR-medium, and DLIR-high). For each reconstruction, two double-blinded radiologists counted up to a maximum of ten metastases. Visibility and contour definitions were also assessed. Comparisons between methods for continuous parameters were performed using mixed models.

**Results:**

A higher number of metastases was detected by one reader with DLIR-high: 7 (2–10) (median (Q₁–Q₃); total 733) versus 5 (2–10), respectively for DLIR-medium, DLIR-low, and ASiR-V (*p* < 0.001). Ten patents were detected with more metastases with DLIR-high simultaneously by both readers and a third reader for confirmation. Metastases visibility and contour definition were better with DLIR than ASiR-V.

**Conclusion:**

DLIR-high enhanced the detection and visibility of liver metastases compared to ASiR-V, and also increased the number of liver metastases detected.

**Critical relevance statement:**

Deep learning-based reconstruction at high strength allowed an increase in liver metastases detection compared to hybrid iterative reconstruction and can be used in clinical oncology imaging to help overcome the limitations of CT.

**Key Points:**

- Detection of liver metastases is crucial but limited with standard CT reconstructions.
- More liver metastases were detected with deep-learning CT reconstruction compared to iterative reconstruction.
- Deep learning reconstructions are suitable for hepatic metastases staging and follow-up.

**Graphical Abstract:**

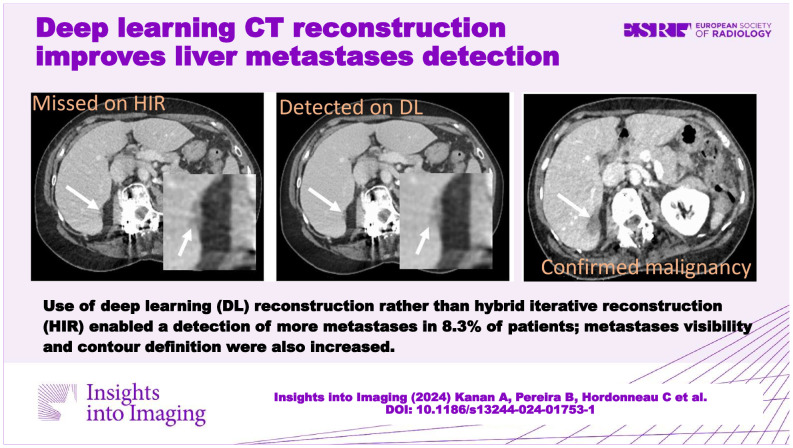

## Introduction

Early detection of liver metastases plays a major role in management options and long-term prognosis and mostly relies on CT [1].

To obtain images from raw data, various algorithms can be used, with iterative reconstructions being the most widespread. Adaptive statistical iterative reconstruction-V (ASiR-V) (GE-HealthCare®) is a hybrid iterative reconstruction technique used in conjunction with filtered back projection in variable proportions according to user preferences. The measured value of each pixel is re-estimated and compared to an ideal predicted through an algebraic noise model. This process is repeated until there is concordance between the estimated and ideal values, thereby reducing noise while maintaining image quality [2].

Detection of liver metastases can be challenging, and CT through hybrid iterative reconstruction methods has certain limitations. It has been shown that using a high percentage of ASiR can lead to a lower image quality, giving it a plastic appearance or an unusually blurry texture, which limits the potential for noise reduction [3]. When comparing CT to MRI, 10% of liver metastases from pancreatic ductal carcinoma were missed [4], and up to 32% were noted as indeterminate [5]. Furthermore, CT scan has a low sensitivity for detecting lesions smaller than 10 mm [6].

Deep learning-based reconstructions are now available and aim to significantly reduce image noise [7, 8]. Deep learning image reconstruction (DLIR) TrueFidelity (GE-HealthCare®) is a new reconstruction method based on a convolutional neural network. The network was trained on thousands of high-quality CT datasets from patients and phantoms, acquired using filtered back projection. It enhances the raw data from a low-dose protocol by comparing it to the optimal data obtained during the training phase. Parameters such as noise, low-contrast resolution, and texture are analyzed and compared. The differences between the two datasets are minimized to achieve the best possible image. This process has been optimized through a learning phase [9]. Three selectable deep learning strength levels (DLIR-low, DLIR-medium, and DLIR-high) are configured by the manufacturer and available for use by clinicians to provide different amounts of noise reduction without impacting reconstruction speed.

These reconstructions provide high quality abdominal CT at same radiation doses compared to iterative reconstructions [10–12].

Several studies have investigated the benefits of DLIR for hepatic lesions. Jensen et al demonstrated that diagnostic confidence scores for abdominal lesions were significantly higher with DLIR compared to ASiR-V. However, their study included all solid organ lesions and did not specifically target hepatic metastases [13]. Nakamura et al found that DLIR resulted in higher scores for the conspicuity of hepatic metastases compared to adaptive iterative dose reduction 3D (AiDR 3D, Canon Medical System®) [14]. However, they did not assess lesion detection. Singh et al showed that DLIR was equivalent to AiDR for the detection of abdominal lesions in a prospective multi-institutional study [15]. Of the 31 lesions evaluated, only 13 were low-attenuating hepatic lesions, which limits the ability to draw definitive conclusions regarding metastasis detection. Therefore, the impact of DLIR on hepatic metastases detection remains unclear.

We hypothesized that the image enhancement from these new reconstruction techniques could allow an increased detection of liver metastases compared to conventional iterative reconstructions. Our main objective was to compare the number of metastases detected using three different levels of DLIR and a 50%-ASiR-V. Our secondary objective was to compare metastases conspicuity for each reconstruction.

## Methods

This was a retrospective observational single institutional study conducted in our medical imaging department.

### Patient selection

All CT scans of the abdomen and pelvis performed for cancer initial assessment or follow-up between November 2020 and July 2021 were selected for the inclusion process. The inclusion criterion was the presence of at least one hypoattenuating liver metastasis described in the radiology report. Exclusion criteria were the loss of at least one reconstruction (loss of raw data, at least one reconstruction not saved on picture archiving and communication system (PACS)), double energy acquisition, age less than 18 years old, hypervascular metastases, and absence of histopathological proof of cancer.

### Imaging technique and CT reconstructions

CT scans were performed using the same Revolution Evo system (GE-HealthCare®) at 120 kV tube, 160 to 500 mA current range with organ dose modulation, 1.375 pitch, 40 mm detector collimation, 0.70 second rotation time, and 1.25 mm thickness. Iodine contrast material was administered with a basis of 2 mL per kilogram adapted to body weight (mean 93 ± 10 mL; range 80-130 mL) (Xenetix 350, Guerbet or Omnipaque 350) into the cubital vein at an injection rate of 2 mL per second. The acquisition was performed 90 seconds after injection. Volume computed tomography dose index (CTDI_VOL_) and dose length product (DLP) were recorded.

One standard 50%-ASiR-V reconstruction and three deep-learning reconstructions were obtained using the DLIR algorithm TrueFidelity at different strength levels: DLIR-low (DLIR-L), DLIR-medium (DLIR-M), and DLIR-high (DLIR-H). All CT scans were anonymized before analysis.

### CT analysis and lesion detection

Metastases number evaluation and subjective analyses and were performed independently by Reader 1, A.K., with three years of in-training experience in radiology, and Reader 2, B.C., with ten years of experience in abdominal radiology. Readers were blinded to the reconstruction method and the patient past medical history. Both readers received identical and standardized printed instructions before evaluation. All CT scans were randomly split into four equal blocks, each block containing one random reconstruction method by patients (121 scans per block). CT scans were analyzed block by block in a random and different order from July to December 2021. To avoid memory bias, an interval of one month between each block analysis was respected. The evaluation was performed on an Advantage Workstation (AW3.2, GE-HealthCare®). Readers were able to adjust the window (width and level) and use coronal or sagittal sections and minimum intensity projection as desired.

Both readers counted the number of hepatic metastases from 0 to a maximum of 10. In cases where both readers found more metastases with DLIR-H than ASiR-V or vice versa, a third independent radiologist (B.M.), with 11 years of experience in abdominal radiology, blindly evaluated the number of lesions on both reconstructions to confirm or disprove the difference. In case of discrepancies, an unblinded consensus reading was made by the three readers. They used all available data, such as MRIs, previous or subsequent CT scans, and clinical reports to verify the metastatic nature of missed lesions, mainly based on their MRI signal characteristics or size variation over time.

Both readers rated overall image quality, image noise reduction, hepatic metastases visibility and hepatic metastases contour definition using a five-point scale: 1-inacceptable; 2-low; 3-medium; 4-good; 5-excellent, based on their own subjectivity.

### Attenuation measurements

Measurements were performed by reader 1, using an Advantage Workstation. Regions of interest (ROIs) were placed on nine anatomical structures (Table 1) and at the center of one randomly selected hepatic metastasis, avoiding artifacts and irregularities. The ROI was then cloned at the same location for each reconstruction. Image noise (N) was defined as the standard deviation of attenuation in the paraspinal muscle. The contrast-to-noise ratio (CNR) of a structure was calculated as the absolute difference between its mean attenuation and the mean attenuation of paraspinal muscle divided by image noise CNR_a_ = │HU_a_- HU_muscle_│/N, (HU: *Hounsfield unit*).

**Table 1:** Image noise and CNRs of anatomical structures and selected metastases for each reconstruction

	ASiR-V	DLIR-L	DLIR-M	DLIR-H	ROI surface (mm²)	
**Image noise**	16.0 ± 2.8	17.2 ± 3.0	13.7 ± 2.7	10.5 ± 3.2		
**Organs CNRs**						
Paravertebral muscle	*NA*	*NA*	*NA*	*NA*	261 ± 134	
Abdominal subcutaneous fat	9.77 ± 2.2	9.13 ± 2.1	11.47 ± 2.6	5.39 ± 3.9	233 ± 146	*p* < 0.001^a^
Abdominal aorta	5.41 ± 1.9	5.06 ± 1.7	6.33 ± 2.1	8.53 ± 2.9	119 ± 45.7	*p* < 0.001^a^
Spleen	3.45 ± 1.2	3.21 ± 1.1	4.01 ± 1.3	5.42 ± 1.8	346 ± 146	*p* < 0.001^a^
Right hepatic lobe	2.95 ± 1.2	2.73 ± 1.1	3.42 ± 1.3	4.61 ± 1.9	440 ± 185	*p* < 0.001^a^
Left hepatic lobe	2.97 ± 1.2	2.75 ± 1.1	3.44 ± 1.3	4.64 ± 1.9	312 ± 136	*p* < 0.001^a^
**Vessels CNRs**						
Main portal vein	5.79 ± 2.1	5.43 ± 1.9	6.79 ± 2.2	9.16 ± 3.2	113 ± 64.6	*p* < 0.001^a^
Right portal vein	5.82 ± 2.1	5.46 ± 1.9	6.83 ± 2.3	9.21 ± 3.3	82.4 ± 42.1	*p* < 0.001^a^
Left portal vein	5.72 ± 2.1	5.39 ± 2.0	6.73 ± 2.3	9.07 ± 3.4	54.4 ± 32.2	*p* < 0.001^a^
**Metastases CNRs**	3.14 ± 1.4	2.89 ± 1.3	3.56 ± 1.7	4.66 ± 2.3	7.75 ± 1.65	*p* < 0.001^a^

Values are given as mean value ± standard deviation

Image noise was defined as the standard deviation of attenuation in the paraspinal muscle

^a^ Statistical difference was observed in pairwise comparison between each reconstruction (*p* *<* 0.001)

### Statistical analysis

Continuous variables were expressed according to their statistical distribution with mean and standard deviation (SD). Metastasis number was, however expressed as median and interquartile range. An arbitrary limit of 10 lesions was given, and the mean number of lesions seemed less appropriate to be presented as a result. Agreement (between both readers and between reconstruction methods) was assessed using Lin’s concordance correlation coefficient. The results were interpreted in relation to recommendations reported in the literature by Altman: < 0.4: no agreement, 0.4–7: poor agreement, > 0.7: moderate to strong agreement [16]. Comparisons between methods for continuous variables were completed using mixed models that allowed to consider between- and within-patient variability (i.e., subject as a random effect). The normality of residuals from these models was analyzed with the Shapiro‒Wilk test and graphical presentation. When appropriate, a logarithmic transformation of the dependent variable has been applied. Statistical analyses were performed using Stata software (version 15, StataCorp, College Station) by B.P. All statistical tests were carried out based on a two-sided type I error at 5%. Sidak’s type I error correction was applied for two-by-two multiple comparisons between methods.

## Results

### Patient and lesion characteristics

**Fig. 1:**
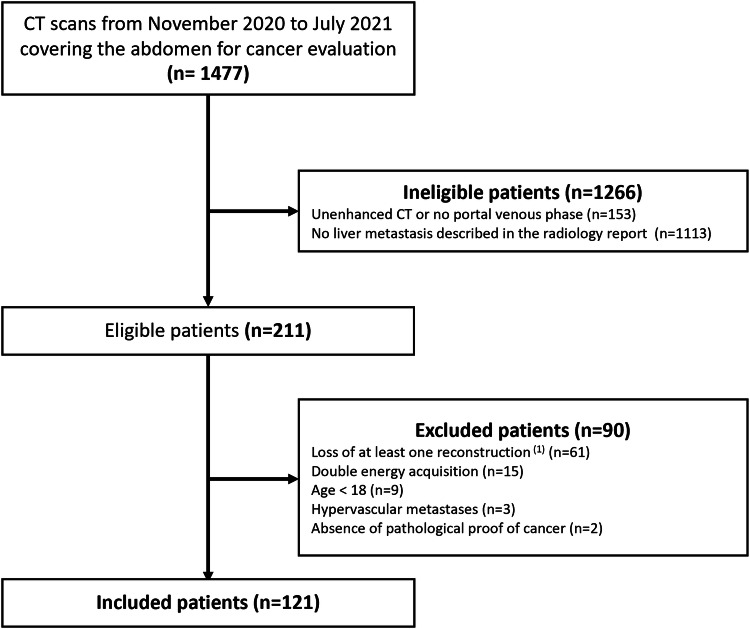
Patient flow-chart. (1) Loss of raw data, at least one reconstruction not saved on PACS

A total of 121 patients were included in the study. A flow chart of the inclusion process is shown in Fig. 1. Patients and primitive tumor characteristics are listed in Table 2.

**Table 2:** Patients and primitive tumor characteristics

Patient characteristics	(*n* = 121) (%)
Demographic	
Male	73 (60)
Female	48 (40)
Mean age, SD	65 ± 12
Cancer type	
Adenocarcinoma	88 (74)
Colic	31 (26)
Pancreatic	29 (26)
Rectal	16 (13)
Gastric	9 (7.4)
Small bowel	2 (1.7)
Ovarian	1 (0.8)
Melanoma	11 (9.1)
Neuro-endocrine	6 (5.0)
Pancreatic	3 (2.5)
Intestinal	2 (1.7)
Hepatic	1 (0.8)
Gastrointestinal stromal tumor	5 (4.1)
Intestinal	4 (3.3)
Gastric	1 (0.8)
Cholangiocarcinoma	5 (4.1)
Ampullary carcinoma	3 (2.5)
Lymphoma	1 (0.8)
Prior systemic treatment ^(a)^	95 (78)

^a^ Prior history of systemic oncologic care, such as chemotherapy and immunotherapy, before CT acquisition

*SD* standard deviation

### Lesion detection

A higher number of metastases was detected by the senior reader (R2) with DLIR-high: 7 (2–10) (median (Q₁–Q₃); total 733) versus 5 (2–10) respectively for DLIR-medium, DLIR-low, and ASiR-V (*p* < 0.001) (Table 3). The junior reader (R1) found no significant difference in metastases number between reconstructions.

**Table 3:** Number of detected hepatic metastases by both readers for each reconstruction

	ASiR-V	DLIR-L	DLIR-M	DLIR-H	
**Reader 1**					
total	673	679	680	686	
median	5 (2–10)	6 (2–10)	5 (2–8)	6 (2–10)	*p* = 0.78^a^
**Reader 2**					
total	686	674	674	733	
median	5 (2–10)	5 (2–10)	5 (2–10)	7 (2–10)	*p* < 0.001^b^

Readers counted up to a maximum of ten lesions per patient

Data are expressed as a total number of lesions with a median per patient (and interquartile range)

^a^ No significant difference was observed between each reconstruction

^b^ Pairwise significant difference was observed between DLIR-H and other reconstructions only

**Fig. 2:**
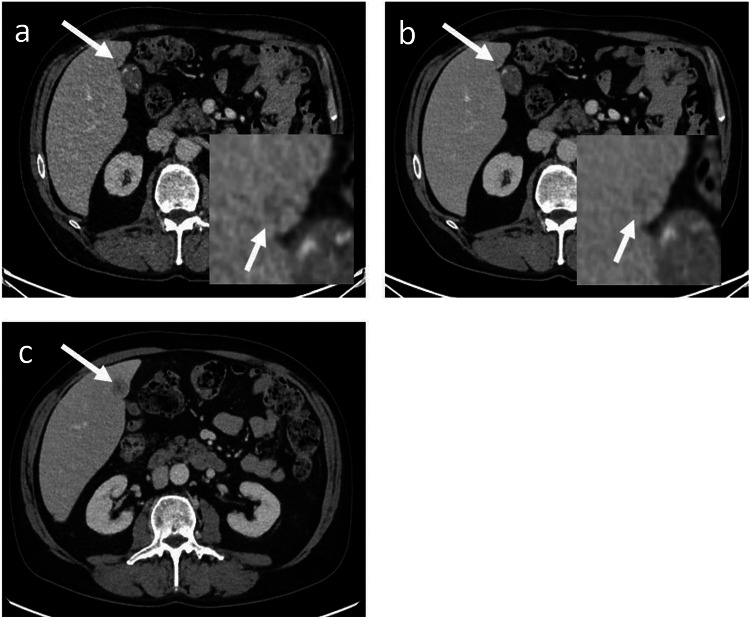
A contrast-enhanced CT image obtained with ASiR-V (**a**) and DLIR-H (**b**) showing the same hypoattenuating metastasis, magnified in the right lower corner (white arrows). Both readers detected the lesion on DLIR-H and missed the diagnosis on ASiR-V, as did a third independent reader. CT image of the same patient two months later showing the growth of the lesion and confirming its malignancy (white arrow) (**c**)

**Fig. 3:**
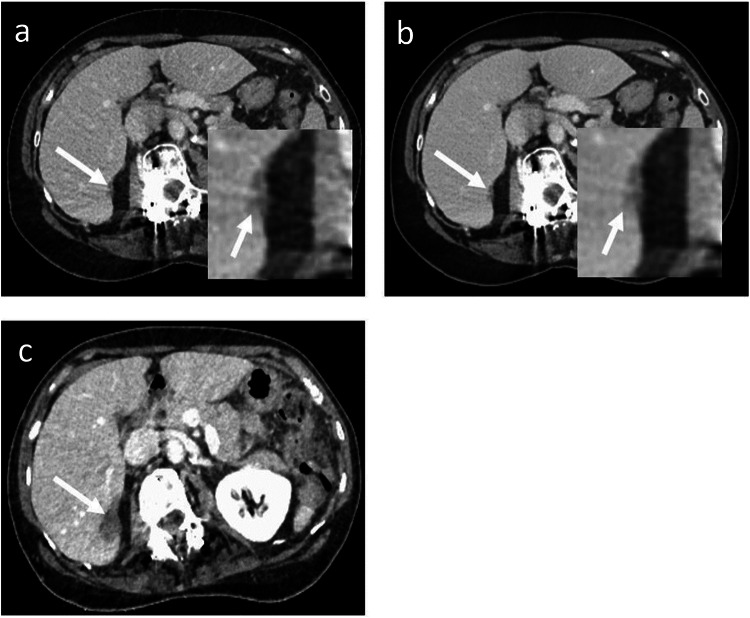
A contrast-enhanced CT image obtained with ASiR-V (**a**) and DLIR-H (**b**) showing the same hypoattenuating metastasis, magnified in the right lower corner (white arrows). Both readers detected the lesion on DLIR-H and missed the diagnosis on ASiR-V, as did a third independent reader. The artifact reduction provided by DLIR can be seen in this example with osteosynthesis material artifact near the lesion significantly reduced. CT image of the same patient 18 months earlier before systemic treatment confirms lesion malignancy (**c**)

For 12 patients, both readers simultaneously found a higher number of metastases with DLIR-H compared to ASiR-V. This was confirmed for ten patients by the third radiologist and disproved for the other two patients (Figs. 2 and 3). Consensus reading was allowed by comparison with subsequent MRI for three patients, subsequent CT for three patients, and previous CT for four patients. In these cases, one additional metastasis was detected using DLIR-H in six patients, and two in four patients. This led to 14 missed lesions with a median size of 7 mm. (details available in Appendix 1).

**Fig. 4:**
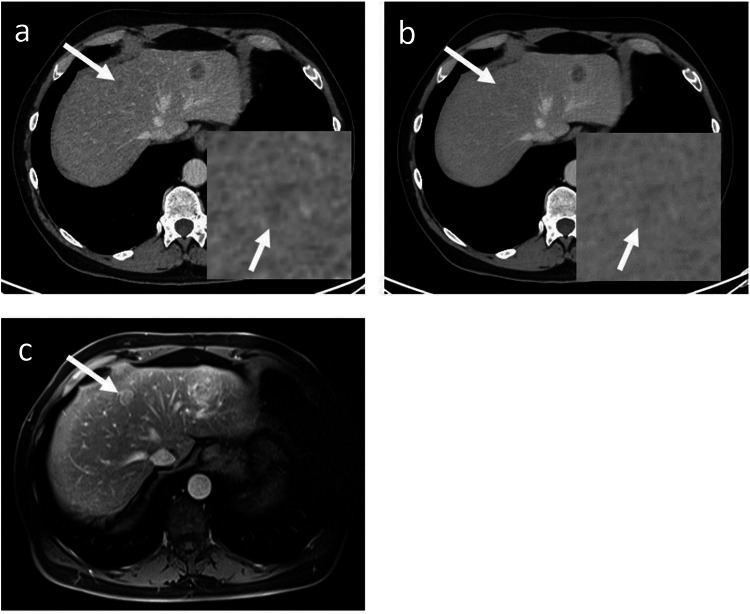
A contrast-enhanced CT image obtained with ASiR-V (**a**) and DLIR-H (**b**) showing the same hypoattenuating metastasis of 11 mm, magnified in the right lower corner (white arrows). Both readers missed the lesion on DLIR-H but not on ASiR-V. MRI of the same patient six weeks later in portal venous phase T1-weigthed image showing lesion growth and confirming its malignancy (white arrow) (**c**)

For two patients, both readers detected a higher number of metastases with ASiR-V than DLIR-H. Confirmation with the third radiologist was obtained for one patient only with consensus reading using a subsequent MRI. The missed lesion was 11 mm in size (Fig. 4).

### CT subjective analysis

**Fig. 5:**
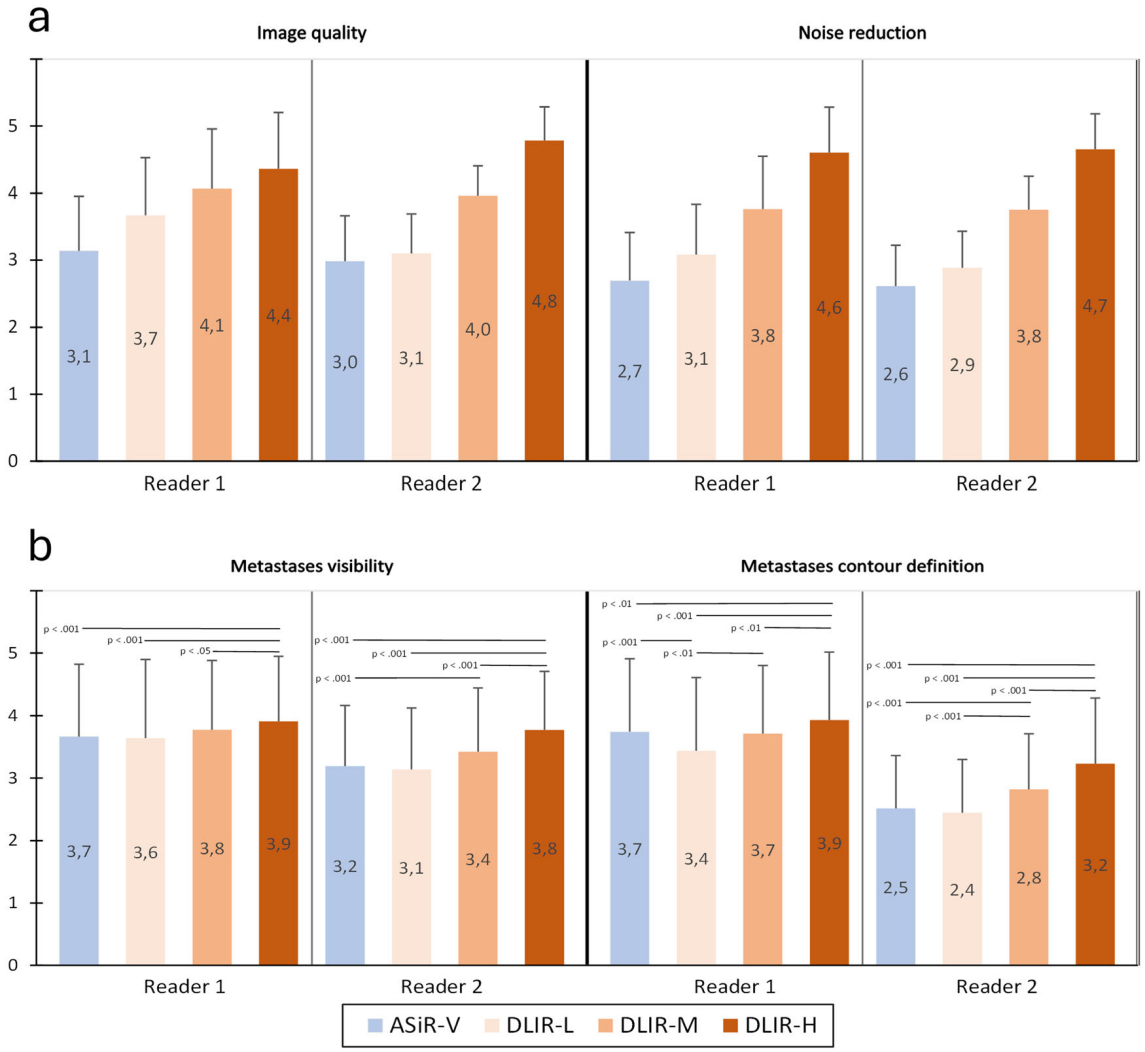
**a** Subjective evaluation of CT image quality and noise. Statistically significant differences were obtained for all pairwise comparisons. **b** Subjective evaluation of hepatic metastases. All statistically significant differences of pairwise comparisons are displayed along with their *p*-values. Values are given as mean score (bars) ± standard deviation (error bars) of a five-point rating scale

Image quality and noise reduction were lower for ASiR-V and increased with deep learning levels (Fig. 5a). Significant differences were observed between all reconstructions for both readers (*p* < 0.001). Metastases visibility and contour definition were better for DLIR-H compared to other reconstructions for both readers (Fig. 5b).

### Attenuation measurements

Image noise was significantly higher for DLIR-L, followed by ASiR-V, DLIR-M, and DLIR-H (*p* < 0.001). CNRs of anatomical structures were significantly different between all reconstructions, with the highest values for DLIR-H followed by DLIR-M, ASiR-V and DLIR-L (*p* < 0.001). CNRs of metastases were higher using DLIR-H and DLIR-M compared to ASiR-V (*p* < 0.001) *(*Table 1*)*.

### Radiation dose

Mean CTDI_VOL_ and DLP were 9,2 mGy ± 2,5 and 512 mGy.cm ± 158, respectively.

## Discussion

This study aimed to compare a recent deep learning-based reconstruction (TrueFidelity) and a standard iterative reconstruction (50%-ASiR-V) for the detection of hypoattenuating liver metastases on CT. The main objective was to determine whether DLIR would affect the number of detected lesions. High-strength DLIR led to a statistical increase in the number of detected lesions for one of the two readers. Additionally, high-strength DLIR enabled both readers to simultaneously detect more metastases in ten patients compared to ASiR-V. This statement was confirmed by a third independent reader. As a secondary objective, we compared lesion conspicuity between both reconstructions. The visibility and contour definition of hepatic metastases received better scores with DLIR-high compared to the other reconstructions for both readers.

The most common etiologies of liver metastases arise from the gastrointestinal tract, mainly colorectal and pancreatic cancers [17]. Management of patients depends on the presence of liver metastases. Their number, size, and location can guide clinicians toward curative or conservative techniques. Treatment modalities include resection surgeries, thermoablation procedures, stereotactic radiotherapy, endovascular treatments, or systemic therapies [18]. Many techniques can be used for liver metastases detection as a conjunct to the initial CT staging. MRI seems to be superior to CT scan, especially for < 10 mm lesions [6, 19]. Fluorine-18-fluorodeoxyglucose positron emission tomography/CT (PET/CT) is also very sensitive but has limited performances for small lesions [20]. Other techniques have been evaluated, such as Kupffer-phase imaging in contrast-enhanced endoscopic ultrasonography, with superior results for small left liver metastases [21].

Liver metastases of digestive adenocarcinoma often appear as multiple hypoattenuating nodular lesions. As adenocarcinomas represented 74% of our study population, comparison between reconstructions were performed on a homogeneous pool of lesions. Imaging features may however change, based on histopathological characteristics and may moderate interpretation of the results. For example, desmoplastic reactions around colorectal liver metastases are closely related to peripheral enhancement [22]. Cystic components of primary tumor and severe necrosis can lead to cyst-like hepatic metastases. Other characteristics may be present, such as calcifications in mucinous adenocarcinoma, and peripheral wash-out and hypervascularity in neuro-endocrine tumors [23].

Despite the multiple modalities, CT scan remains the gold standard for gastro-intestinal cancer staging and follow up according to international recommendations. The current protocol often involves a CT scan of thorax, abdomen, and pelvis [24–26]. Obtaining high-quality images is therefore essential and implementation of artificial intelligence-based reconstruction algorithms facilitate early detection of metastases.

Deep learning methods still have certain limitations. Kaga et al showed that high levels of deep learning can reduce the conspicuity of hepatic lesions compared to ASiR-V, especially for small lesions [27]. This was also described for extrahepatic exploration such as chest CT, where small structures had lower conspicuity scores with high-strength DLIR [28]. Yang et al found no difference in liver lesion detection between DLIR and ASIR-V; however, they only involved 8 patients and 13 malignant lesions in their analysis [29].

There have been recent studies experimenting different low-dose protocols of DLIR in terms of quality assessment of images and lesion detection [30–36]. Wang et al found that low-dose deep learning algorithms may provide better images, signal-to-noise, and contrast-to-noise ratios of unenhanced CT scans when compared to standard-dose iterative reconstruction. They found no difference in sensitivity and diagnostic confidence for liver metastases detection [37]. When comparing a 33%-dose protocol with DLIR to a standard-dose iterative reconstruction, Lee et al found lower noise on DLIR and comparable diagnostic performance in detecting malignant liver tumors [38].

The aim of our study was to determine whether diagnostic performance was superior at the same dose level, specifically if more hepatic metastases could be detected. To our knowledge, no study has yet described an increase in the number of detected hepatic metastases using deep learning reconstructions compared to iterative reconstructions. This finding could significantly impact patients’ oncologic evaluation. Our study showed that more metastases were detected in 10 out of 121 patients with DLIR. As expected, the majority of missed lesions were smaller than 10 mm, as subcentimeter lesions can often be missed on CT scans [3]. Detecting these small lesions can significantly influence the therapeutic management of patients, such as surgical or ablative planning in colorectal cancer, or a switch from curative to palliative care in pancreatic adenocarcinoma.

Our study has some limitations. First, this was a retrospective study, relying on a single CT and one manufacturer’s DLIR. Second, our inclusion criterion was solely based on the final CT report with no prior confirmation of our own. However, all cases had histopathological proof of the primary cancer. Most of the patients had previous MRIs, which were used for comparison by radiologists allowing reduction of potential selection bias. We only included patients with liver metastases, which suggests that our results showed DLIR to increase sensitivity rather than specificity for lesion detection. Finally, we did not perform subgroup analyses based on primary tumor type.

Although our study doesn’t evaluate patient management and outcome, our results suggest a potential implication of DLIR and can be considered as an aid in selecting the most appropriate CT reconstruction and the level of deep learning for liver metastases detection. In conclusion, high-strength DLIR statistically increased the detection and conspicuity of liver metastases compared to ASiR-V. Additional studies should be conducted to assess the clinical impact of these findings, but our results encourage the use of deep-learning reconstructions when performing abdominal CT scans in oncology.

### Supplementary information

ELECTRONIC SUPPLEMENTARY MATERIAL

## Data Availability

The CT scans analyzed during the current study are not publicly available to protect study participant privacy according to local legislation. The anonymized analyzed data are available from the corresponding author upon reasonable request.

## Abbreviations

ASiR-V: Adaptive Statistical Iterative Reconstruction-V
CNR: Contrast-to-Noise Ratio
CTDI_VOL_: Computed Tomography Dose Index
DLIR: Deep Learning Image Reconstruction
DLIR-H: Deep Learning Image Reconstruction High
DLIR-L: Deep Learning Image Reconstruction Low
DLIR-M: Deep Learning Image Reconstruction Medium
DLP: Dose Length Product
