# Supplementary material for: Deep learning CT reconstruction improves liver metastases detection
Source: Insights Imaging. 2024 Jul 6;15:167. doi: 10.1186/s13244-024-01753-1 (PMC11227486; doi:10.1186/s13244-024-01753-1)
Supplement: Supplementary file 1 — ELECTRONIC SUPPLEMENTARY MATERIAL [file 13244_2024_1753_MOESM1_ESM.pdf]

Deep learning CT reconstruction improves liver metastases detection

ELECTRONIC SUPPLEMENTARY MATERIAL

Appendix 1. Follow up data of patients with concordent differences between ASiR and DLIR-H simultaneously by both readers for detected metastases and their consensus reading

Detection step: Difference of number of detected metastases (blinded to reconstructions)

	Patient number	Reader 1 (junior) n(ASIR)-n(DLIR-H)	Reader 2 (senior) n(ASIR)-n(DLIR-H)	Reader 3 (senior) n(ASIR)-n(DLIR-H)	Differences between R1 and R2		Missed lesions size1 (mm)	Missed lesions size 2(mm)	Modality used
DLIR-H > ASiR	#9	2	1	1	Confirmed by R3		6	*NA*	subsequent MRI
	#25	1	3	2	Confirmed by R3		7	21	previous CT
	#27	1	1	1	Confirmed by R3		13	*NA*	previous CT
	#40	2	1	2	Confirmed by R3		7	4	previous CT
	#68	1	3	1	Confirmed by R3		7	*NA*	subsequent MRI
	#77	1	4	2	Confirmed by R3		4	5	subsequent CT
	#82	3	5	2	Confirmed by R3		8	7	subsequent CT
	#83	2	2	1	Confirmed by R3		9	*NA*	subsequent CT
	#84	3	2	0	Disproved by R3 (no consensus reading)				
	#104	1	1	1	Confirmed by R3		12	*NA*	previous CT
	#113	2	1	1	Confirmed by R3		8	*NA*	subsequent MRI
	#120	7	4	0	Disproved by R3 (no consensus reading)				
TOTAL NUMBER OF PATIENTS	12				10	2	**14 lesions missed. Median size of missed lesions on ASiR: 7 mm**		
ASiR > DLIR-H	#18	-1	-2	0	Disproved by R3 (no consensus reading)				
	#114	-1	-2	-1	Confirmed by R3		11	*NA*	subsequent MRI
TOTAL NUMBER OF PATIENTS	2				1	1	**One missed lesions on DLIR-H : 11 mm**		

Insights Imaging (2024) Kanan A, Pereira B, Hordonneau C, et al.
